# Newspapers and the Doctors

**Published:** 1893-07

**Authors:** 


### NEWSPAPERS AND THE DOCTORS.

The secular press, as a rule, is decidedly inimical to the regular medical profession. The reason assigned by the profession is, that regular physicians do not advertise. Be that as it may, the large majority of newspapers show a decided preference for the advertising class, and seldom have a good word for the class who do not advertise. Of course, there are exceptions; but even the exceptions may be accounted for on personal grounds. It may be that an editor, or a local editor or reporter, is especially friendly to a doctor, or may be under obligations to him. He may feel that he ought to say something nice about this doctor, —and he goes ahead and says it. It may be, doubtless is, well-meant; these parties do not understand all the nice points in medical etiquette, and know little or nothing of our code, and hence it happens, oftener than otherwise, that the editor or reporter says something (intended, mind you, for a compliment or as a help to the doctor) of which the doctor, when he sees it, is very much ashamed; something, may be, which makes him ridiculous in the eyes of his colleagues, or even brings him into contempt with his brethren;—besides, in many cases, the doctor is unjustly suspected of being privy to the "notice."

So well known is the fact that one is often suspected in matters of this kind, that physicians have, in some localities, requested editors to please not mention their name in connection with any "case." That, we think, is going a little too far. In case of an accident, it is the newspapers' business to give the particulars, and, we think, legitimate, to say Dr. So and So set the bone, for instance, or rendered timely surgical or medical aid.

But this thing takes a wide range, and it is hard to draw a line. If there is one thing which is held in contempt by the regular profession more than another, it is the indirect method of advertising; getting one's name in the paper in connection with every accident or case of serious illness; and we may say, rightly or not, the one owning the name is very promptly and very generally suspected of it, if it occur often, may be accused of it; and we know of more than one instance where it has been brought up against a member in his local society. Readers may recall a quite notorious case, which led to the expulsion of a prominent member of a County Society, and it turned out in the end that the offensive paragraph had been inserted in the paper by the father of a grateful patient, without the knowledge, even, of the physician named.

Not very long ago, a newspaper stated that Doctor ——, in a lower county, had bought another pair of fine horses; that his practice was so extensive and laborious that he kept six horses in steady and active use, etc. This paragraph was promptly clipped by another doctor in the same town, and sent to the editor of the JOURNAL. The doctor referred to by the newspaper disclaimed authority for its appearance, and said he had often asked the editor to not use his name in any such way.

The following case has recently been brought to the JOURNAL'S

attention. It must be very exasperating to the physician with whose name such liberty is taken, for he is a well known member of the State Association and of several local Societies, and is generally respected as an ethical physician. It is hardly to be supposed that he would authorize a statement which many physicians in his county know to be not true; and to make such claim for him is sure to bring him into contempt with his professional brethren of the very best class. A paper which will take such a liberty with a physician as to really scandalize him, as in this case, should be prosecuted for libel. We reproduce the article, and a comment on it by X. Y. Z., in another paper, suppressing the physician's name, but giving the paper. The JOURNAL will be pleased to have this question discussed:

"Dr. B. was called to —— this week, to perform the sixth operation of intubation ever performed in the State, he having performed the other five. The patient was a little child of Mike Friste, suffering with diphtheritic croup, and its life was despaired of when the doctor reached it. In half an hour the child was playing, and suffering no inconvenience from the operation. The doctor has the only case of instruments of the kind in the State. 'Es ist emer goor etwas suwezen.'"*

"EDITOR FORUM:—Referring to the above, please allow me to say the *Times* is mistaken. The operation of intubation has not only been performed by other physicians in the State of Texas besides Dr. B., but it has been performed thirteen times by a physician residing in the city of T—. It is not owing to ignorance or lack of skill on the part of the profession, that this opertion is not performed oftener; it is because it is considered no safer than tracheotomy, the death rate being about the same from each operation."—*X. Y. Z., in Forum.*

If anything were lacking to make completely absurd the position in which the doctor is placed by this doubtless well meant paragraph, it is the fact, as we have been informed, that the child, so opportunely saved, (?) died shortly after the operation; to say nothing of the German proverb which assumes for Dr. B all the medical knowledge of the State. [Name and place given in the "*Times*," but omitted here.—ED.].

---

THE PATHOLOGICAL MUSEUM OF THE UNIVERSITY OF TEXAS. —The Medical Department of the University of Texas is exceedingly anxious to collect an many unique or typical examples of pathological processes as possible, for purposes of illustration in

*It is good sometimes to know something."

